# An intersectional gender analysis in kidney transplantation: women who donate a kidney

**DOI:** 10.1186/s12882-021-02262-9

**Published:** 2021-02-16

**Authors:** Laura Rota-Musoll, Serena Brigidi, Esmeralda Molina-Robles, Ester Oriol-Vila, Laureano Perez-Oller, Mireia Subirana-Casacuberta

**Affiliations:** 1 Department of Nephrology, Consorci Hospitalari de Vic, Vic, Catalunya Spain; 2 Research group on Methodology, Methods, Models and Outcomes of Health and Social Sciences (M3O), Faculty of Health Science and Welfare, Centre for Health and Social Care Research (CESS), University of Vic-Central University of Catalonia (UVIC-UCC), Vic, Spain; 3 Department of Anthropology, Philosophy and Social Work in the University of Rovira i Virgili (URV), Tarragona, Catalunya Spain; 4 Department of Nursing Management, Parc Taulí Health Corporation Consortium, Sabadell, Catalunya Spain

**Keywords:** Gender, Intersectionality, Kidney donor, Living donor transplantation, Qualitative methodology

## Abstract

**Background:**

Living-donor transplantation is the best treatment option in patients with chronic kidney failure. Global data show that women are less likely to be kidney recipients than men but are more likely to become living kidney donors. We explored the experience of women who donate a kidney to relatives with biological and socio-cultural ties and to understand the similarities and differences in their experience.

**Methods:**

A qualitative hermeneutic phenomenological study with an intersectional analysis of gender. Ten women donors accepted in the transplant evaluation period participated, all of whom donated a kidney to a pre-dialysis relative. Two categories were included: women with biological kinship ties (mothers, sisters) and women who have a socio-cultural relationship (wives) with kidney recipient. The data were collected through semi-structured in-depth interviews and analysed using thematic analysis.

**Results:**

Women donate their kidneys in a convinced manner, without worrying about their health, with an optimistic and positive attitude, and without believing that they are acting heroically. Women with biological kinship ties see it as a ‘naturalization thing’. In contrast, wives donate conditioned by gender roles, but also as a form of empowerment and as a personal benefit: they donate in order to avoid taking on carer role for their husband and as a way of protecting their children.

**Conclusion:**

The study’s findings expand the conception of kidney donation as solely altruistic and may help professionals to pay attention to the complexity and intersectionality of features present in women who are living kidney donors.

## Introduction

According to the literature, the population with end-stage-renal disease (ESRD) that needs renal replacement therapy (RRT) is increasing [1]. Further, access to this therapy is uneven depending on the region, with only 50% of those who need RRT receiving treatment [2].

Living-donor kidney transplantation is the best treatment option for survival and quality of life outcomes of patient with kidney disease [3]. In 2017, 36% of kidney transplants worldwide were from living donors [4]. Kidney donation is considered a safe procedure, although there are risks in the perioperative process [5] and studies have shown that donors have a slightly higher risk of cardiovascular and long-term kidney disease than the healthy population [6–8]. In general, living kidney donors have psychosocial outcomes like quality of life that are the same or better after the donation and in comparison with non-donors [5]. Living donor may have to bear a financial burden (uncovered financial expenses and lost salary) [9, 10] and difficulty organising their life during the donation period [11].

Chronic kidney disease shows differences between men and women in prevalence [12] and rate of progression [13, 14]. However, in different parts of the world there is unequal access to and possibilities for renal replacement treatment between men and women [15, 16]. There are also differences in access to kidney transplantation. Women are less likely than men to receive a cadaveric kidney transplant [17]. In some cases, women had discussions, on fewer occasions than men, with health professionals about kidney transplantation as a therapeutic option [18]. In others, testing was finished too late to be included in the cadaver donor list [19].

Regarding living-donor kidney transplantation, gender disparity is very clear: women donate more kidneys than they receive [14, 20, 21]. Official global data show that 6 out of every 10 kidney donors are women [22].

### Literature review

Kidney donation has gender. Several studies show that gender inequality in transplants favours men, while women end up donating more than they recieve and even selling their kidneys [23, 24]. Women seem to be more willing than men to volunteer and take risks in living donation [25]. There is no conclusive evidence as to why women donate more and receive fewer kidneys [21]. Some studies that have analysed the reasons behind this disparity show that socioeconomic factors play an important role. Donating a kidney leads to economic loss at several levels depending on the conditions of each country [26, 27]. As a result, some studies have suggested that different income levels and work situations between men and women may influence this disparity [21]. Others point out that women may be driven to donation owing to ideological discourses that consider men the main providers of material resources within families [28, 29]. The social context in which this research takes place, gender system, as a structuring of societies based on relations of privilege and power, leads to an unequal access to economic, social and symbolic resources [30]. Furthermore, healthcare field is highly feminised [31]. Within this framework, there are studies in the social sciences that provide an alternative explanation to altruism, which for years has been the basis for organ donation, showing that kidney donation by women may be explained as a form of social reproduction [32], or as a result of social pressure (economic dependence and care burdens) [24, 33–35].

Some studies specify that it is mostly mothers, followed by wives, that donate kidneys [36]. One metanalysis shows that the closer the relationship between people, the greater the tendency to donate [37]. Some studies highlight that the decision to donate a kidney should be seen in the context of the network of close family relationships [38] and that the moral roles of kinship and the ambiguities in family relationships influence the decision to donate within the family [23, 32, 37–40].

The aim of the study is to explore the experiences of two categories of women who donate their kidneys in the family context: women with biological kinship ties with the recipient (mothers, sisters) and women with social-cultural ties (wives) and examine whether there are similarities and differences in their experiences.

## Materials and methods

### Approach and design

This study uses the method of hermeneutic phenomenology following the postulates of Heidegger [41]. Hermeneutic phenomenology explores lived experience in depth, with the goal of creating meaning and understanding [42]. This interpretive approach made it possible for us to capture the essence of the women donors’ experiences during the kidney donation process.

We feel it is important to analyse living-donor kidney transplantation from a gender intersectional perspective since, as feminist theory proposes [43, 44], including the category of gender, as a social construction, provides us with a more complete level of analysis of the differences and inequalities in health between men and women.

Study manuscript is based on the consolidated criteria for reporting qualitative research (COREQ) [45] (see Table 1).

**Table 1:** Consolidated criteria for reporting qualitative studies (COREQ): 32-item checklist

Domain	Item No	TopicTopic	Guide Questions/Description	Reported on Page No.
**Research team and reflexivity**				
Personal Characteristics:				
	1	Interviewer/facilitator	Which author/s conducted the interview or focus group?	Page 3: “One of the women authors (LR), who is not part of transplant team, contacted each of participants and went to their homes to conduct the interviews”.
	2	Credentials	What were the researcher’s credentials?	N/A
	3	Occupation	What was their occupation at the time of the study?	N/A
	4	Gender	Was the researcher male or female?	Page 3: “Six researchers (five women and one man) participated in this study”.
	5	Experience and training	What experience or training did the researcher have?	Page 4: “Four of the researchers (LRM, SB, EMR, MSC) had previous experience in qualitative study designs”.
Relationship with participants:				
	6	Relationship established	Was a relationship established prior to study commencement?	Page 3: “One person in charge from each kidney transplant unit identified participants who met inclusion criteria and informed them of the study.”
	7	Participant knowledge of the interviewer	What did the participants know about the researcher?	Page 3: “One of the women authors (LR), who is not part of transplant team, contacted each of participants”.
	8	Interviewer characteristics	What characteristics were reported about the interviewer/facilitator?	Page 12: “Participants received verbal and written information about main researcher and study aims”.
**Study design**				
Theoretical framework:				
	9	Methodological orientation and Theory	What methodological orientation was stated to underpin the study?	Page 2: “This study uses the method of hermeneutic phenomenology following the postulates of Heidegger [40]”.
Participant selection:				
	10	Sampling	How were participants selected?	Page 3: “A purposive sample was used”.
	11	Method of approach	How were participants approached?	Page 3: “One person in charge from each kidney transplant unit identified participants who met inclusion criteria and informed them of the study. Once they had given verbal consent, one of the women authors (LR), who is not part of transplant team, contacted each of participants”.
	12	Sample size	How many participants were in the study?	Page 3: “A total of 10 participants agreed to participate in the study. All the participants had biological kinship (30% mothers or sisters) or sociocultural (70% wives) ties”.
	13	Non-participation	How many people refused to participate or dropped out? Reasons?	Not aplicable-All who contacted participated in the study.
Setting:				
	14	Setting of data collection	Where was the data collected?	Page 3: “one of the women authors (LR), who is not part of transplant team, contacted each of participants and went to their homes to conduct the interviews and sign the written consent”.
	15	Presence of non-participants	Was anyone else present besides the participants and researchers?	Page 3: “One or more individual interviews were held for each participant, before and after the donation, depending on the stage of donation process the women were in during the period of the study”.
	16	Description of sample	What are the important characteristics of the sample?	Table 2
Data collection:				
	17	Interview guide	Were questions, prompts, guides provided by the authors? Was it pilot tested?	Page 3: “Interview script arose from a review of the literature related to the purpose of the study, as well as from the work done with fellow kidney transplant professionals. Script was previously tested with three female donors that had already gone through the kidney donation process”.
	18	Repeat interviews	Were repeat interviews carried out? If yes, how many?	Page 3: “Data saturation was achieved in the 17 interviews conducted with 10 participants.” Page 3: “One or more individual interviews were held for each participant, before and after the donation, depending on the stage of donation process the women were in during the period of the study”.
	19	Audio/visual recording	Did the research use audio or visual recording to collect the data?	Page 3: “Interviews were recorded and transcribed verbatim”.
	20	Field notes	Were field notes made during and/or after the interview or focus group?	Page 3: “Field notes were taken in each interview that were retrieved during the data analysis stage”.
	21	Duration	What was the duration of the interviews or focus group?	Page 3: “Average length of the interviews was 36 min (between 26 and 53 min)”.
	22	Data saturation	Was data saturation discussed?	Page 3: “Data saturation was achieved in the 17 interviews conducted with 10 participants.”
	23	Transcripts returned	Were transcripts returned to participants for comment and/or correction?	Page 3: “Participants were given the interview transcript for comment”.
**Analysis and fidings**				
Data analysis:				
	24	Number of data coders	How many data coders coded the data?	Page 4: “Data analysis was reviewed by two team members“.
	25	Description of the coding tree	Did authors provide a description of the coding tree?	N/A
	26	Derivation of themes	Were themes identified in advance or derived from the data?	Page 3: (Data Analysis) “We then proceeded to generate initial codes and organise the topics and sub-topics that arose inductively”.
	27	Software	What software, if applicable, was used to manage the data?	N/A
	28	Participant checking	Did participants provide feedback on the findings?	Not applicable as participants did not provide feedback on the data/findings.
Reporting	29	Quotations presented	Were participant quotations presented to illustrate the themes / findings? Was each quotation identified?	Page 4–9 (Results): Quotations are presented throughout the text alongside interpretations.
	30	Data and findings consistent	Was there consistency between the data presented and the findings?	Page 4–11 (Results and Discussion)
	31	Clarity of major themes	Were major themes clearly presented in the findings?	Page 4–9 (Results)
	32	Clarity of minor themes	Is there a description of diverse cases or discussion of minor themes?	Page 4–11 (Results and Discussion)

*From: Tong A, Sainsbury P & Craig J. Consolidated criteria for reporting qualitative research (COREQ): a 32-item checklist for interviews and focus groups Qual Assur Health Care. 2007;19(6):349–357*

### Participants

Study participants were adult women who donated kidneys to pre-dialysis recipients.

Inclusion criteria were female donors accepted for kidney donation in three kidney transplant units in Barcelona. Recipients had to be at least 18 years of age with chronic renal failure that had not begun dialysis treatment. This more restrictive inclusion criterion is important for the study authors since whether the recipient had or had not initiated dialysis could influence the experience of the women donors. Study was carried out between November 2017 and February 2020. Six researchers (five women and one man) participated in this study.

This study is part of a larger prospective study where donor-recipient pairs are selected and studied from the donor evaluation process to 6 months after surgery. A total of 10 participants agreed to participate in the study. Their characteristics are shown in Table 2. All the participants had biological kinship (30% mothers or sisters) or sociocultural (70% wives) ties.

**Table 2:** Characteristics of the participants

Participant	Age	Relationship to the recipient_**5**_	Sex of the recipient
**D1**	71	Wife	Man
**D2**	64	Wife	Man
**D3**	74	Wife	Man
**D4**	66	Wife	Man
**D5**	52	Wife	Man
**D6**	57	Wife	Man
**D7**	44	Mother	Woman
**D8**	58	Mother	Man
**D9**	60	Mother	Man
**D10**	70	Sister	Woman

### Data collection

Data were obtained through semi-structured in-depth interviews. Interview script arose from a review of the literature related to the purpose of the study, as well as from the work done with fellow kidney transplant professionals. Script was previously tested with three female donors that had already gone through the kidney donation process.

A purposive sample was used. One person in charge from each kidney transplant unit identified participants who met inclusion criteria and informed them of the study. Once they had given verbal consent, one of the women authors (LR), who is not part of transplant team, contacted each of participants and went to their homes to conduct the interviews and sign the written consent. One or more individual interviews were held for each participant, before and after the donation, depending on the stage of donation process the women were in during the period of the study. Average length of the interviews was 36 min (between 26 and 53 min). Interviews were recorded and transcribed verbatim, and field notes were taken in each interview that were retrieved during the data analysis stage. Participants were given the interview transcript for comment. Data saturation was achieved in the 17 interviews conducted with 10 participants.

### Data analysis

A thematic analysis [46] was performed in order to understand meanings produced by the stories of the women that donate a kidney to a relative. Interviews were first read and re-read while both listening to audio recording so as not to miss any subtle information and writing down notes in the margins [47]. We then proceeded to generate initial codes and organise topics and sub-topics that arose inductively. All the relevant data for each topic were collected, and the link between these and the research objectives was verified. Next step in the analysis process involved verifying and contrasting findings by returning to original text and reworking topics that would enable us to understand meanings of the women kidney donors. Data analysis was reviewed by two team members. Four of the researchers (LRM, SB, EMR, MSC) had previous experience in qualitative study designs.

## Results

All women donated their kidneys to men, except for two biologically related donations that went from mother to daughter and between sisters (see Table 2).

Through thematic analysis we identified five topics:

### THEME 1: convinced decision-making

Women’s decision to donate a kidney to a relative was taken to improve life and health of the recipient. Participants had witnessed recipient’s illness from the beginning or for at least 10 years. Seeing recipient’s health deteriorate and limitations appear in his/her life influenced the decision to donate. All donations were before the recipient needed dialysis. Making donation to avoid dialysis treatment was one of participants motivations.

Decision was also easy and clear. Women comment that although they can share situation with family and friends, decision was intimate and personal.

“I went for it! I said: First me, and if that isn’t possible, we’ll look for someone”. (…) “I decided 5 years ago and I haven’t regretted it once”. (D5_1).

All the participants volunteered to be donors, although they had never considered being organ donors before. In no case did the recipient ask them. According to interviews, the decision to donate a kidney was made free of external pressure and internal doubts.

*“I was very clear about it. I never felt any obligation to donate”* (D9_1).

Women stated that they felt convinced and confident in themselves and the decision they had made. After the donation, at no time did they regret the decision to donate and would do so again.

### THEME 2: not worrying about their health

Women donors perceived their good health prior to donation, which was confirmed in the results of examinations and physical tests during the evaluation process, which showed they were healthy and could donate a kidney.

Participants considered that their health after the post-surgery recovery period would be the same and they were not worried about possible complications or their long-term health. When asked about their future health, they said they were not afraid of changes or alterations in their health and trusted the good results of kidney transplantation and the health systems and its professionals.

*“I see my health after the donation as the same as now, I don’t notice any differences”* (D4_1).

They were also not worried about donating an organ as they had no interest in keeping their body intact.

Once the donation was made, neither long-term health nor organ loss concerns appeared in the women donors.

The only change in participants’ health was aimed at maintaining their good health in order to be in the best condition for the donation. Since they were accepted as donors, they recognised that they took greater care of themselves, in sense of changing unhealthy habits: they improved some eating habits or gave up drinking alcohol or smoking. In every case we saw that the women donors increased their physical exercise, increasing the frequency or starting a new sports.

### THEME 3: showing an optimistic and positive attitude

Participants showed an optimistic and positive attitude about many aspects of donation. They acknowledged that the evaluation period for being a kidney donor was intense and long and sometimes difficult to reconcile with their work and personal obligations, but it did not become a burden.

They went to surgery with a calm and confident attitude that there would be no complications either for them or for the recipient. They thought that surgery would be successful and that the donation would go well. Despite knowing about the possible complications in receiving the organ, they did not think about recipient rejection.

*“There’s no reason why something should happen. Everything will remain the same”.* (D7_1).

Dealing with health professionals during the donation process and the good overall results of living-donor kidney transplantation helped them maintain a positive attitude.

Women donors maintained a positive attitude before and after the donation.

### THEME 4: donation is not a heroic act

All participants agreed that donating a kidney is not a heroic act. They refused to believe that donating an organ was so important. Neither before nor after the donation, did they see it as something that should be praised and required great recognition.

“*People say: ‘Oh, what a great thing you did’. I don’t! I see it as completely normal. I don’t see it as making a great sacrifice or as a big deal, no. I see it as normal”.* (D5_1).

“*People make a big deal, a very big deal about it. And I think: it’s not that big a deal”* (D6_1).

Before kidney donation, study participants had played an important supporting and caring role with family.

“I am family mainstay because I’ve had to keep things going for a long time. I looked after my father, then my mother, my uncle and aunt. I’ve always had to be there. And in my house everyone trusts me a little”. (D9_1).

There is a difference between donors with biological kinship ties and those that have a sociocultural relationship with the recipient. Former saw donation as “a naturalization thing”. Many used the term ‘normal’ in their accounts: donation within the family is conceived as a normal.

“*When they talked about the need to a transplant, her father and I went immediately to do the tests and it turned out that I could give it to her. (…) I see that she is happy [and thinks] ‘Mother can do it’. In the family, you know? (…) I said to her: if all goes well, I gave you life and now I will give it back to you*”. (D3_1).

In contrast, for the donor wives, the situation they experienced with the progression of their husbands’ illness and its consequences in their lives influenced donate decision.

“*The situation takes you there. You enter a world that you don’t know how you got there and you have to continue”* (D9_1).

“*You never thought you would be in a situation where perhaps one day you’d have to donate an organ*” (D6_1).

“*Many people say: ‘Oh, you are really brave! I don’t know if I would do it’. That’s not the point. You have to find yourself in the situation and then decide”* (D6_1).

Wives also said that donation was seen as “doing what is expected”, both from their own and others’ perspective. In the above quotes we can see how the women were convinced in their decision. Within the family, the news of this decision that they made freely and clearly was not experienced as a conflict but was accepted.

“*Mum, you have done a good thing*” (D2_1).

*“I think it was a good deed and I did it for him” (D2_2).*

“*I did a good thing*” (D6_2).

The donation of the wives makes sense and is part of the moral relationship of couple.

“*I think I should do it. I should do it for him. He’s my partner and he’s the person I will live with*”. (D7_1).

“*He would also do it*”. (D10_1).

Most of wives said that gender-differentiated education influenced their decision to give:

*“I don’t think it’s a genetic thing, but rather that they educate us in the values of caring for others and that makes us more willing to do it”.* (D6_3).

*“Women see beyond, think more of others”.* (D9_1).

### THEME 5: personal benefits: avoiding carer role and protecting the children

Experiencing the donation as a personal benefit was found in the group of donor wives. It meant that participants not only experienced satisfaction in helping a loved one who needed a kidney, but also emotional and personal benefits for themselves:

“*When you have the chance to do something for somebody, I wouldn’t feel good not doing it!”.* (D6_1).

“*I feel bad if I see that he’s feeling bad*”. (D7_1).

*“There’s a part that wants him to be well, but there’s also a selfish part that thinks that if he isn’t well I won’t be either. Our life would change a lot (…) I want you to be well so that our life being well is extended as long as possible”.* (D6_1).

Personal benefits experienced by wives was in no way related to the type of relationship that would arise after donation, since the donor wives expected that the relationship with the recipient would remain the same, unchanged. And so, it was for the women who made the donation; they claimed that the relationship with their husbands had not changed.

Wives were well aware of prognosis of chronic kidney disease and knew that without a kidney transplant their husbands would end up on dialysis, which would greatly affect the quality of life and that of their families. Donating the kidney to their partners would mean avoiding taking on carer role for their husbands.

“If he gets sick, what? *I don’t want to take care of a sick person*” (D2_1).

“I don’t want to have two kidneys and have to take care of him” (D6_3).

Another personal benefit that women with a sociocultural relationship mentioned was that they felt satisfied in having been accepted as donors to their husbands since this ruled out their children being possible donors. Donating the kidney to their husband protected their children from becoming kidney donors for their parents.

*“My son found it very difficult to understand. He said to me: ‘I’ll give it to dad’. And I said to him: ‘No! You will not give it to him’. No! I didn’t want it to be him. No!”* (D8_1).

*“The first thing my children said was: ‘Mum, you are very old, we’ll give it to dad’. And I said no! I’d prefer to give it to him myself. We’re not going to live as long as them. And they need both kidneys, you never know what life may bring ….”* (D10_1).

## Discussion/Conclusion

This study provides knowledge from a gender perspective on the similarities and differences between the experiences of women who donated a kidney to relatives with biological kinship ties (children and siblings) and women who donated a kidney to their husbands.

The main reasons why they decided to donate a kidney are the same for all the women. All of them witnessed the recipient’s illness. The desire to improve their health and prevent them from entering dialysis were key to their decision-making. Concern for the recipient health and the wish to improve his/her quality of life as well as avoid complications or even death were reported in previous studies [37, 48–51]. Participants decisions in our study were made with ease and conviction, in line with previous studies [48, 49].

With regards to the donor’s health, they were aware of their good health and, according to Manera et al. [52] this is important when deciding to donate a kidney. Participants were optimistic and confident about donation and their future health. Gill [53] found that hope and optimism are coping mechanisms that help with the stress triggered by living-donor kidney transplantation. Acording with the literature [48, 54, 55] the donors were more worried about recipient health that their own short- and long-term health. Thus, healthcare professionals need to provide more information on the risks that donors run.

Women expressed no desire to keep their body intact. Some studies [48, 56, 57] find that after donation donors do not experience any sense of loss with regards to donated organ.

Like other studies [49, 58, 59], participants did not consider the donation to be a heroic act. One contribution of our study was to find differences between women when delving more deeply into this aspect. Moreover, as other studies [38, 60], participants with biological kinship bonds said that they saw their donation as a ‘natural thing’, as the natural consequence of family bonds, as a matter of course within the family. We understand this normality as a social construction that is framed in a certain context [61]. In this case the donation takes place within the family, which is where, in Sahlins [62] terms, mutuality of being is developed, conditioned by values and moral obligations that bind people [63].

Wives decision needs to be understood within the network of conjugal relations, but it is also influenced by gender roles constructed in the social context in which they live. Women who donated a kidney to their husbands had not previously thought of being organ donors, and it was the context of their spouse’s illness and the moral roles within the couple that influenced their decision. Ummel and Achille [56] argue that genetically or emotionally related donors are driven to donate their kidney due to their moral obligations within the family.

One of the study’s findings, consistent with others [28, 32], is that the gender role could have influenced women to offer themselves as donors for their husbands. All the women participants had previously provided care and assistance to members of their family (children, parents, mother or father-in-law). Subsequently, these women not only donated their organ, but also took care of recipient at home after surgery. We can compare this care service with other types of care work, such as caring for the elderly or sick relatives, which is undertaken disproportionately and in a “normalized manner” by women [64] and that, in consequence, affects their health and quality of life [65, 66]. It is not possible to generalise from our small sample but, as Zeiler [33] states, it would be an ethical concern if gender disparity in living-donors were explained by traditional roles that force women to care for the sick members of their family. Wife participants of our study were aware that gender discourse and education influenced their decision to donate a kidney to their husband. According to our findings, gender-differentiated education may be an ally for organ donation.

However, wives stated that the donation brought them personal benefits for themselves. They experienced emotional benefits that went beyond improving the recipient’s life and that were related to mitigating feelings of sadness and frustration they experienced on seeing their husband’s suffering and not doing anything for him. These feelings of frustration have been observed in people who have been rejected as kidney donors [67].

Wives knew that without the kidney transplant the autonomy of their husbands would be more limited and that they would need care and assistance. Kidney donation meant they could avoid taking on the role of carer for their husbands. This act of empowerment and control over their lives coincides with De Groot’s study [68], where women donors thought that the transplant allowed recipient to be less dependent and more involved in family life, thus improving donors’ quality of life.

This research we found that, unlike other studies where women could donate because of structural and economic pressures [29, 34, 69], wives donated their kidneys as a personal benefit and as a form of empowerment. It is essential that healthcare professionals pay attention to these aspects in order to better accompany the women’s decision-making with regards to kidney donation.

To finalize it is important to highlight that has not been established in the literature. With the decision to donate, women who donate to their husbands protect their children by ruling them out as possible donors. This is how they play a protective role towards the younger members of the family.

The study has some limitations. All the participants volunteered for the donation. To learn more about donation and gender it might be interesting to delve more deeply into the experiences of women who did not volunteer to donate a kidney to a family member with kidney failure and learn about their thoughts and motivations. At the same time, men’s motivations to donate should also be explored**.** Furthermore, we cannot generalize our data to donors in countries where access to kidney replacement treatment is very different from Spain. Future research is required to clarify all the factors that could play a key role in gender disparity in living-donor kidney transplantation.

## Conclusion

Women donate their kidneys with clarity, without concern for their own health, with an optimistic and positive attitude and without believing that they are committing a heroic deed. Women with biological kinship ties to recipient see the donation as a ‘naturalization thing’. In contrast, wives donate conditioned by gender roles but also do it as a form of empowerment and as a personal benefit: they donate in order to avoid taking on carer role for their husband and as a way of protecting their children.

Study examines the gender gap in donation and provides new insights into and a better understanding of the life of women who donate a kidney.

The study’s findings expand the conception of kidney donation as solely altruistic and can help professionals pay attention to the complexity that women living kidney donors face.

## Data Availability

The datasets generated and/or analysed during the current study are not publicly available to protect the identification of the participants but are available from the corresponding author on reasonable request.
